# Uncertainty Quantification and Sensitivity Analysis for the Electrical Impedance Spectroscopy of Changes to Intercellular Junctions Induced by Cold Atmospheric Plasma

**DOI:** 10.3390/molecules27185861

**Published:** 2022-09-09

**Authors:** Jie Zhuang, Cheng Zhu, Rui Han, Anna Steuer, Juergen F. Kolb, Fukun Shi

**Affiliations:** 1 Suzhou Institute of Biomedical Engineering and Technology, Chinese Academy of Sciences, Suzhou 215163, China; 2 School of Biomedical Engineering (Suzhou), Division of Life Sciences and Medicine, University of Science and Technology of China, Hefei 215000, China; 3 Leibniz Institute for Plasma Science and Technology (INP), 17489 Greifswald, Germany

**Keywords:** cold atmospheric plasma, uncertainty quantification, sensitivity analysis, Cole-Cole model, intercellular junctions

## Abstract

The influence of pertinent parameters of a Cole-Cole model in the impedimetric assessment of cell-monolayers was investigated with respect to the significance of their individual contribution. The analysis enables conclusions on characteristics, such as intercellular junctions. Especially cold atmospheric plasma (CAP) has been proven to influence intercellular junctions which may become a key factor in CAP-related biological effects. Therefore, the response of rat liver epithelial cells (WB-F344) and their malignant counterpart (WB-ras) was studied by electrical impedance spectroscopy (EIS). Cell monolayers before and after CAP treatment were analyzed. An uncertainty quantification (UQ) of Cole parameters revealed the frequency cut-off point between low and high frequency resistances. A sensitivity analysis (SA) showed that the Cole parameters, *R_0_* and *α* were the most sensitive, while *R_inf_* and *τ* were the least sensitive. The temporal development of major Cole parameters indicates that CAP induced reversible changes in intercellular junctions, but not significant changes in membrane permeability. Sustained changes of *τ* suggested that long-lived ROS, such as H_2_O_2_, might play an important role. The proposed analysis confirms that an inherent advantage of EIS is the real time observation for CAP-induced changes on intercellular junctions, with a label-free and in situ method manner.

## 1. Introduction

Cold atmospheric plasma (CAP) has been investigated and applied intensively in numerous biomedical fields, such as wound healing, treatment of oral and skin diseases, and cancer therapy [1,2,3]. Studies have shown that the reactive oxygen and nitrogen species (RONS) generated by CAP are the main factors inducing subsequent biological responses, e.g., alterations in membrane permeability, integrity of cytoskeleton, structural stability of DNA, mitochondrial functions and intercellular junctions [4,5].

The intercellular junctions, pervasive in biological tissues and multicellular systems, play a key role in CAP-related biological effects [6]. Generally, intercellular junctions consist of tight junctions, adherens junctions, and gap junctions, etc. These junctions are not only responsible for maintaining the structural integrity of the tissue, but also essential for the communication and transmission of signals and substances between cells [7]. Tight junctions are multi-protein complexes composed of transmembrane and cytoplasmic protein, which control the molecular flow between cell layers and may affect the permeability of RONS [8]. Adherens junctions are primarily responsible for the assembly between cells and extracellular matrix (ECM) by connecting intercellular gaps with actin cytoskeleton, through the transmembrane cadherin family [9]. Gap junctions are mainly composed of connexins which control the electrical and metabolic (sugars, ions, amino acids, nucleotides) communication functions [10]. Therefore, investigating the effect of CAP on the intercellular junctions of cell monolayers can deepen our understanding of interactions between CAP and multicellular systems.

A multitude of studies have shown that CAP can manipulate intercellular junctions of epithelial cells, keratinocytes, fibroblasts and so on. Schmidt et al. found that the expression of the gap junctional protein connexin 43 in keratinocytes was downregulated 1 h after CAP treatment with kINPen 11 by means of Western blotting and RT-PCR, indicating that the gap junctions were broken [6]. Through immunofluorescence staining, Hoentsch et al. found that CAP can disrupt tight junctions in murine epithelial cells (mHepR1), characterized by the retraction of zonula occludens protein (ZO-1) from cell membrane [11]. Furthermore, Haertel et al. observed a decrease of the expression of E-cadherin in HaCaT-keratinocytes after 30 s treatment with kINPen 09 by means of fluorescence labeling, which proved that adherens junctions could be disrupted by CAP [12,13]. In addition to the above mentioned direct impact, a recent study showed that RONS produced by CAP were also transmitted from treated cells to untreated cells through intercellular junctions, resulting in a bystander effect [14]. Notably, most of the relevant studies about CAP treatments on intercellular junctions relied on diagnostic methods that are typically invasive, label required, cytotoxic and time consuming [6,8,11]. Electrical techniques can prevent related issues. For instance, trans-epithelial electrical resistance (TEER) measures the ohmic resistance of cell layers via direct current or low frequency alternating current in order to identify alterations in the integrity of intercellular junctions [15]. Nonetheless, this method is highly dependent on the electrode configuration and insensitive to changes in cell or cell monolayer morphology [16,17].

In previous studies, by exposing epithelial cells to nanosecond pulsed electric fields (nsPEFs), we concluded that electrical impedance spectroscopy (EIS) is suitable for the characterization of nsPEF-induced changes in cell membrane and intercellular junctions [17,18,19]. EIS obtains the frequency-dependent electrical properties of an analyte by applying a low-voltage, broad-bandwidth AC signal, thereby providing a real-time, non-invasive, label-free, in situ manner to continuously monitor changes in cell structures and functions over time [18,20,21]. Moreover, bioimpedance plays an essential role in discriminating the responses of normal and malignant cells to physicochemical stimuli [22].

The Cole-Cole model is a widely used method in impedimetric analysis, featuring excellent universality, simplicity, and fast computation in fitting, and is appropriate for real-time online monitoring [23]. However, the Cole parameters suffer from poor repeatability and ambiguous biophysical significance [17]. Examining the influence of model input uncertainties on model output can, on the one hand, improve the accuracy of model prediction and, on the other hand, determine the parameter sensitivity in order to optimize the fitting computation [24].

Therefore, uncertainty quantification (UQ) and sensitivity analysis (SA) are anticipated to improve the accuracy of the Cole-Cole model and evaluate the link between the Cole parameters, thereby aiding in the determination of the corresponding biophysical significance. The propagation of input uncertainties to outcomes were investigated first to increase the comprehension of the model and to distinguish the most sensitive parameters for optimizing the fitting procedure. Next, the temporal development of critical Cole parameters was analyzed to gain insight into the dynamic changes of intercellular junctions in normal and malignant cell monolayers after CAP treatment. Accordingly, this study aims at investigating the temporal development of a normal rat liver epithelial cell line (WB-F344) and its malignant counterpart (WB-ras) after exposure to a non-lethal dose of CAP-treatment, utilizing EIS combined with subsequent impedimetric analysis based on the Cole-Cole model. Concurrently, the respective analysis was improved by UQ and SA. To the best of our knowledge, yet no study has employed EIS to examine the interactions between CAP and intercellular junctions. The findings may provide a novel methodology and some new experimental data for elucidating the mechanisms of how CAP affects intercellular junctions.

## 2. Results and Discussion

### 2.1. UQ and SA of the Cole-Cole Impedance Model

#### 2.1.1. Propagation of Model Input Uncertainties to Outcomes

**Figure 1:**
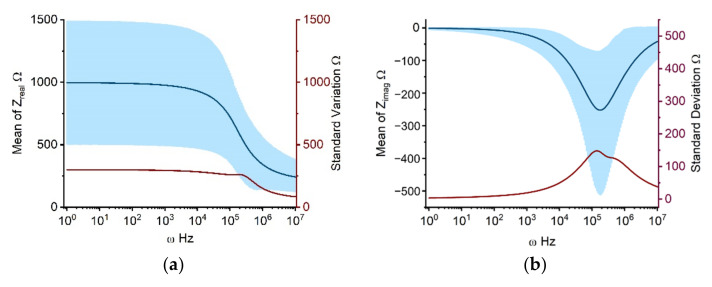

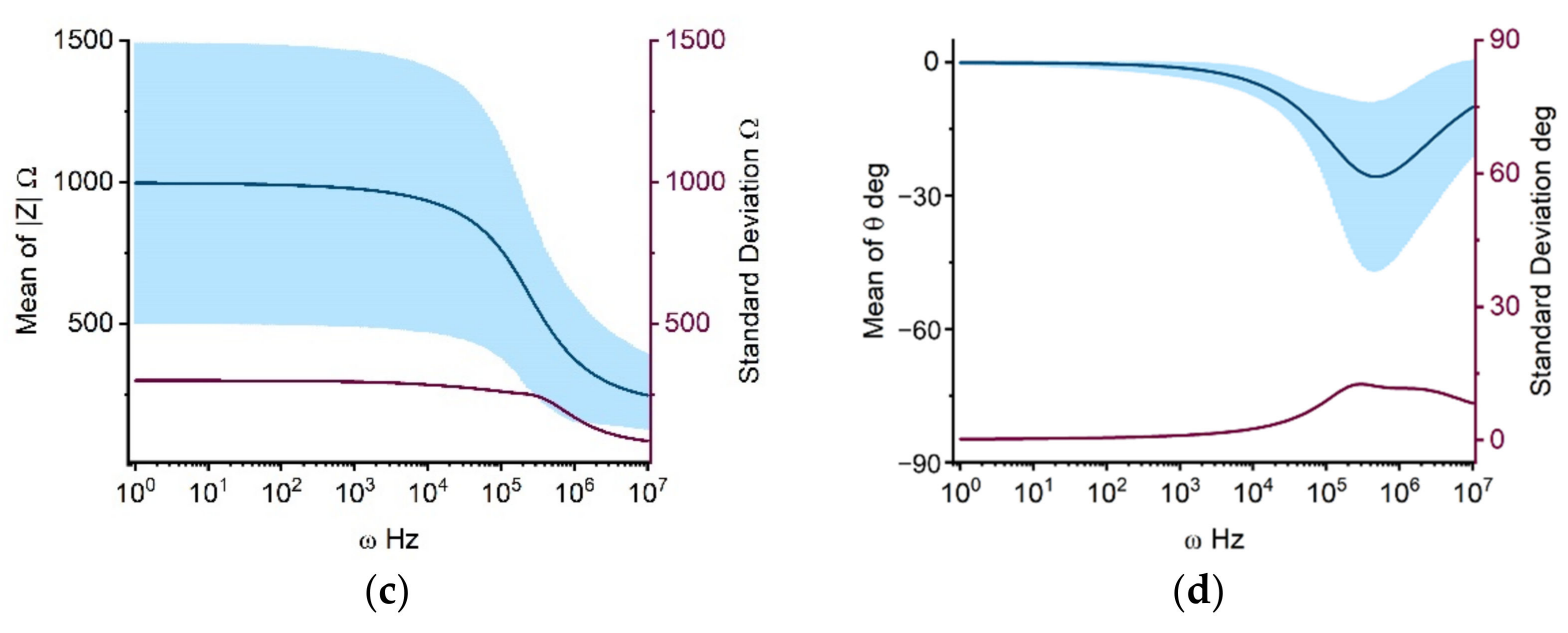
UQ quantifies the mean, standard deviation, and 90 percent prediction space of real (**a**), imaginary (**b**), magnitude (**c**), and phase (**d**) of the Cole impedance taking input uncertainties into account. The solid dark blue line depicts the mean value with frequencies ranging from 1 Hz to 10 MHz. The dark red line and light blue area represent the standard deviation and 90% prediction space, respectively.

Individual impedance components, calculated using the Cole-Cole model with parameters in Table 1, are presented in Figure 1. Figure 1a depicts the real part of the Cole impedance. The value is nearly constant at 1000 Ω from 1 Hz to 10 kHz. Beginning at 10 kHz, the impedance begins to fall progressively, approaching 300 Ω at 10 MHz. This stepwise impedance decrease from 10 kHz to 10 MHz is mostly attributable to β-dispersion due to the interfacial polarization at the cell membrane, which is in accordance with the results of various earlier investigations [17,18]. Figure 1a shows that the standard deviation of the real part of the impedance remains essentially around 300 Ω from 1 Hz to 200 kHz, then drops stepwise from 200 kHz to 10 MHz, to a minimum of 100 Ω. In Figure 1a, the upper and lower bounds of the 90% prediction space have a similar shape as the real component of the impedance curve, with the upper limit decreasing from 1500 Ω at 10 kHz to 400 Ω at 10 MHz and the lower limit decreasing from 500 Ω at 10 kHz to 200 Ω at 10 MHz. Figure 1b shows the imaginary part of the Cole impedance, in which the frequency-dependent curves of mean values, standard deviation, and 90% prediction space, all exhibit a bell shape. The highest amplitude is observed at 200 kHz. Figure 1c,d illustrate the amplitude and phase of the Cole impedance, which has a similar tendency as the real part (Figure 1a) and the imaginary part (Figure 1b) of the Cole impedance, respectively.

From the UQ results for the Cole impedance in Figure 1a,b, it is clear that the high uncertainties in the model output are concurrent with large mean values. The real part of the impedance represents the resistive characteristics of the cell monolayer, which increase as frequency decreases. The imaginary part of the impedance, on the other hand, is related to the capacitive characteristics of the system with its maximum value occurring approximately at 200 kHz, which coincides with the steepest section of the real part curve. This relationship exists as a result of the fulfilment of the Kramers-Kronig relationship for the real and imaginary impedance [18,25]. The frequency-dependent standard deviation and 90% prediction space of the Cole-Cole model output can assist determining the frequency of interest. For example, guidance can be offered for studies employing single-frequency impedance as indices, which is crucial for reducing the complexity of measurement hardware in real-world applications [26]. Taking individual differences and measurement mistakes into account, the excessive standard deviation and 90% prediction space suggest that the impedance at the corresponding frequency may include a considerable error as a single-frequency index for system changes. For the impedance curve shown in Figure 1, the impedance between 10 kHz and 100 kHz may be a feasible solution with less errors as the index of change for a specific system. The amplitude and phase of the Cole impedance are consistent with the variation of the real and imaginary parts, respectively, because the absolute value of the real part of the impedance is significantly greater than that of the imaginary part within 200 kHz. Therefore, while determining the amplitude, the real part determines the primary variation. In the phase calculation, the real part of the impedance is employed as the denominator to reduce the phase amplitude within 10 kHz. Around 200 kHz, the real and imaginary parts of the impedance become closer, and the phase value increases, with the imaginary component bearing the most influence. In following analysis, we focus on the real and imagined parts of the impedance.

#### 2.1.2. Parameter Sensitivity for the Real and Imaginary Part of Cole Impedance Outcomes

**Figure 2:**
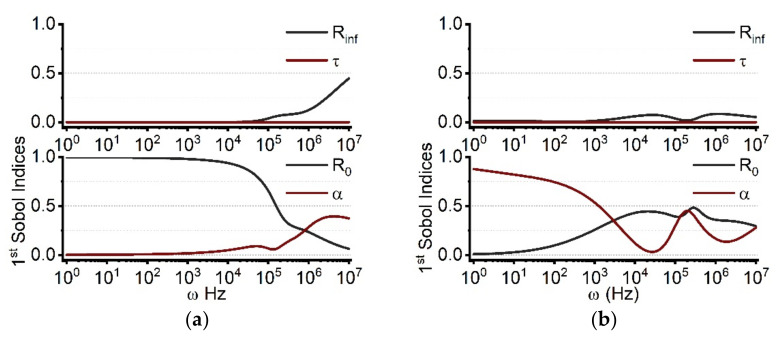
The parameter sensitivity distributions for the real (**a**) and imaginary (**b**) parts of the Cole impedance. The low frequency for the real part is determined by *R_0_*, while *α* amplifies the influence in the high frequency range. On the contrary, *α* mainly determines the low frequency for the imaginary part, and *R_0_* primarily determines the high frequency.

As depicted in Figure 2, we computed the influence of uncertainty for individual Cole parameters (*R_0_, R_inf_, α, τ*) on the real and imaginary parts of the cell monolayer impedance. The goal was to quantify the sensitivity of each parameter to the model output. The frequency range is set from 1 Hz to 10 MHz. The *y*-axis quantifies the first-order Sobol index, which varies from 0 to 1 and represents the contribution percentage of the corresponding parameter to the model output. Figure 2a shows the parameter sensitivity for the real part of the impedance. In the low frequency region (*f* < 50 kHz), *R_0_* is the dominant factor with a value close to 1. As frequency increases, the impacts of *α* and *R_inf_* begin to increase while the effect of *R_0_* declines progressively. In comparison to the other characteristics, *τ* has a negligible effect throughout the entire frequency range. At frequencies beyond about 1 MHz, the influence of *α* on the model output exceeds that of *R_0_* by rising up to 0.45, while *R_0_* falls to 0.1 for10 MHz. At 10 MHz, the influence of *R_inf_* rises from 0 at 10 kHz to 0.5 at 10 MHz. Figure 2b demonstrates that the parameter sensitivity for the imaginary part of the impedance has significantly different frequency-dependent changes than the real part of the impedance. When the frequency is less than 1 kHz, *α* is the most important parameter. After 10 kHz, the Sobol index of *α* eventually falls below 0.5 and exhibits an oscillating pattern as the frequency increases. The impacts of *R_inf_* and *τ* are less than 0.1 across the whole frequency range (1 Hz–10 MHz).

The sensitivity distribution of the Cole parameters, correlated to the imaginary part of the impedance, exhibits significant differences, as seen in Figure 2. *R_inf_* and *R_0_* play dominant roles at high and low frequencies of the real part, respectively. The boundary between low and high frequencies of the bioimpedance spectrum, which is typically ambiguous, can be determined via SA analysis. The Sobol index of *R_inf_* is 0.2 at 2 MHz, while that of *R_0_* is 0.19. As frequency increases, the influence of *R_inf_* increases and the influence of *R_0_* decreases. Hence 2 MHz can be identified as the boundary between low and high frequencies when a single Cole-Cole model is satisfied. Another sensitive parameter is *α*, denoting the dispersion width [27,28]. The dispersion width is related to the multiple time scales of dielectric relaxation processes in a system [18,27]. The SA demonstrates that the impedance around 1 MHz is relatively more sensitive to various polarization components. At different frequencies, the amplitude of the imaginary part is alternately governed by *α* and *R_0_*. The low frequency is primarily impacted by *α*, whilst the high frequency is primarily determined by *R_0_*. Since the imaginary part of the impedance is related to the capacitive properties of the system, i.e., dielectric relaxation, the influence of *α* will be amplified. Overall, SA can aid in determining the measurement range and simplify the fitting process.

**Figure 3:**
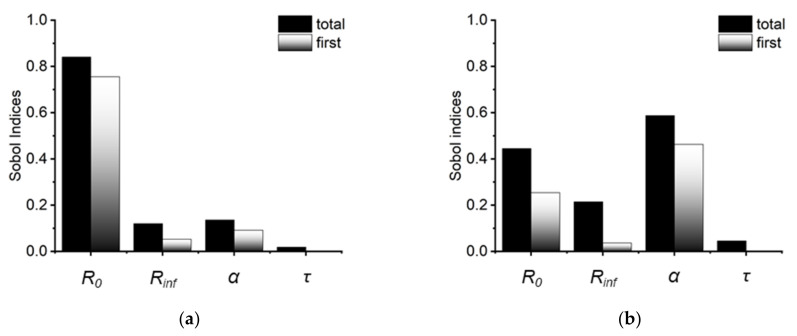
The first order and total Sobol indices of Cole parameters for the real part (**a**) and imaginary part (**b**) of the Cole impedance. Larger total Sobol indices than first order Sobol indices indicate interactions among the Cole parameters.

As shown in Figure 3, the first-order and total Sobol indices for each Cole parameter over the entire frequency range were calculated. Since an impedance measurement is accomplished primarily through logarithmic frequency point sampling, the data points in the low frequency region are denser, and the average Sobol indices of the more sensitive parameters at low frequencies will increase accordingly. In Figure 3, it is evident that the total Sobol indices are greater than the first-order Sobol indices in all four Cole parameters. The latter only evaluates the impact of one parameter on the output of the model. The former encompasses both the influence of a single parameter and the interaction between two parameters on the model output. When these two are not equal, it implies that the parameters have an interaction relationship [29]. This interaction between parameters might rise to the problem of poor fitting repeatability. We believe that this interaction is the origin of the ambiguity of the Cole parameters. Consequently, when using the Cole-Cole model to repeat trials, the less sensitive parameter can be determined once and then fixed to conserve computer resources for subsequent calculations. In most cases, the CAP treatment had negligible effect on the media conductivity. Hence, *R_inf_*, which is a low-sensitivity parameter, can be fixed to accelerate the calculation and improve fitting accuracy.

### 2.2. Temporal Development of the Cole Parameters Extracted from Cell Monolayer Impedance after CAP Treatment

As indicated in Section 3.3, in situ continuous impedance measurements were performed for both WB-F344 and WB-ras cell monolayers before and after CAP treatment. In this work, one single impedance acquisition took less than 7 s. After CAP treatment, impedance measurements continued for up to 24 h with a minimum 1-min gap between measurements. The impedance data of CAP-treated cell monolayers were then evaluated based on the Cole-Cole model with UQ and SA. In this experiment, the resistance at high frequency of all treated groups was nearly identical to that of untreated control. This may be attributed to the fact that short term CAP treatment did not considerably alter the conductivity of the media. Therefore, the value of *R_inf_* was fixed, as calculated from the control group, in following fitting procedures for all groups.

#### 2.2.1. Normalized Low Frequency Resistance (*R_0_*) Related to Intercellular Junctions

**Figure 4:**
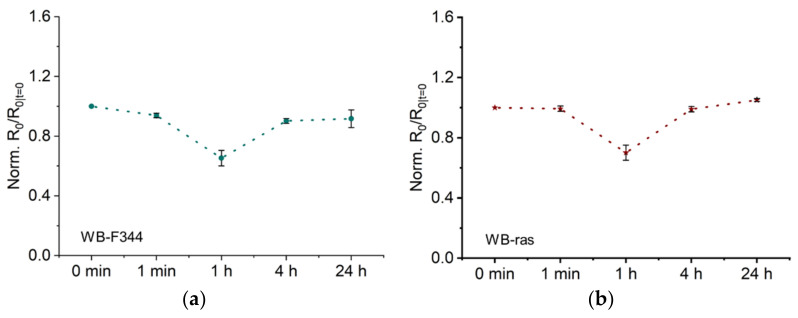
Low-frequency resistance *R_0_* as a function of time after CAP treatment of two cell monolayers, WB-F344 (**a**) and WB-ras (**b**).

Figure 4a,b illustrate the temporal development of *R_0_*, obtained by fitting the impedance spectra of WB-F344 and WB-ras cell monolayers to the Cole-Cole model, after CAP treatment from 1 min to 24 h, respectively. To facilitate comparison, *R_0_* values of both cells were normalized to that of the untreated control. As shown in Figure 4a, *R_0_* of the WB-F344 cell monolayer decreased observably 1 min after CAP treatment. One hour later, it fell to 65% of the untreated level. Four hours later, measurements revealed a considerable recovery in *R_0_* back up to 0.9, which remained almost constant after 24 h. Figure 4b depicts the temporal development of *R_0_* for the WB-ras cell monolayer, showing a similar profile of change to its normal counterpart. There was no notable decline in *R_0_* at 1 min, but it became higher than that for WB-F344 at 1 h and afterwards; e.g., *R_0_* at 24 h has reached 1.05.

In our previous research on the same cells, membrane electroporation, changes in intercellular junctions and cell morphology were the primary factors influencing *R_0_* [17]. Disruption of intercellular junctions, such as adhesion junctions and tight junctions, can significantly decrease *R_0_*. Conversely, cell swelling, caused by the imbalance in osmotic pressure between the interior and the exterior of cells after onset of electroporation, could significantly increases *R_0_*. In this investigation, there was no substantial increase in *R_0_* after CAP treatment, and the plasma jet did not come into direct contact with cell monolayers. Therefore, it is reasonable to rule out the existence of CAP-induced electroporation. The results of this work are in line with previous reports that the electric field in the plasma jet plume is insufficient to induce electroporation [30].

As stated above, the substantial decrease in *R_0_* indicates that intercellular junctions of cell monolayers were changed after CAP treatment. Numerous earlier investigations have reached similar conclusions. Hoentsch et al. discovered that a kINPen plasma treatment for only 30 s significantly altered the adhesion capability of mouse epithelial cells (mHepR1), and led to the degradation of the tight junction protein ZO-1. These findings suggest that CAP can damage cell adhesion junctions and tight junctions [11]. Haertel et al. discovered that calmodulin E (E-cadherin) expression of keratinocytes (HaCat) was dramatically decreased and cell adhesion junctions were significantly disrupted following a 30-s kINPen treatment [12]. In addition, experimental and simulation evidence suggests that CAP can inhibit Cx43 (the primary gap junction protein)-related mRNA and protein expression and disrupt the structure of gap junctions [6,14]. For both cell monolayers, the value of *R_0_* was nearly fully restored after 4 h, indicating that damaged intercellular junctions were repaired. The effect of non-lethal CAP treatment on intercellular junctions is therefore reversible, consistent with a previous report. In that work, J. Choi et al. discovered that CAP led to the downregulation of E-calmodulin expression and prevention of intercellular junction formation, and that these alterations were completely reversed within 3 h [13]. Notably, the EIS method employed in this study offers a distinct benefit over existing research in that it permits quick, label-free, continuous in situ monitoring of CAP-induced changes in cell monolayers.

#### 2.2.2. Normalized Dispersion Width (*α*) Related to Extracellular Space

**Figure 5:**
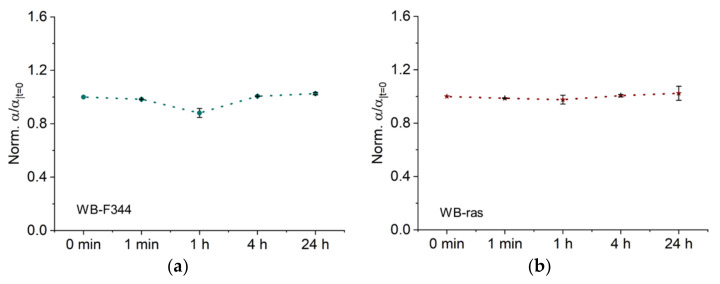
Dispersion width *α* as a function of time after CAP treatment of two cell monolayers, WB-F344 (**a**) and WB-ras (**b**).

Figure 5 demonstrates the temporal development of normalized Cole parameter α, which represents the dispersion width. As shown in Figure 5a, *α* of WB-F344 decreased significantly to 0.88, 1 h after CAP treatment. After 4 h, values for *α* recovered and remained there even after 24 h. In contrast, *α* of WB-ras did not change significantly at any time point after CAP treatment (Figure 5b), demonstrating that CAP had a greater impact on WB-F344 cells than on WB-ras cells in terms of changes of *α*.

The dispersion width, *α*, is generally considered related to the extracellular space and can be used to quantify the tortuosity. For instance, Ivorra et al. conclude that changes of *α* were associated with the morphology of the extracellular space [28]. In short, a decrease of *α* means an increase of tortuosity of the extracellular space [27]. In a prior work, we found that the tortuosity of the extracellular space is closely related to the presence of tight junctions. [17]. After one hour of CAP treatment, *α* of WB-F344 decreased by 12% in this study. We therefore hypothesize that CAP treatment had an influence on the tight junctions of WB-F344, whereas *α* of WB-ras remained nearly unaltered, indicating that its tight junctions were not susceptible to the CAP parameters used in this investigation. This phenomenon may be attributed to the fact that normal cells have more and tighter tight junctions than their malignant counterparts. As a result, normal cells are more sensitive to CAP stimulation and their alterations can be more pronounced. This is consistent with the fact that WB-F344 and WB-ras have distinct *R_0_* values. In a prior work, the outcomes of 100-ns pulsed electric field treatment of WB-F344 and WB-ras cells were compared using the Cole-Cole model. WB-ras cells demonstrated a smaller α change than WB-F344 cells [31], which partially supports the hypothesis above from another perspective.

#### 2.2.3. Normalized Characteristic Time Constant (*τ*) Related to Membrane Capacitance

**Figure 6:**
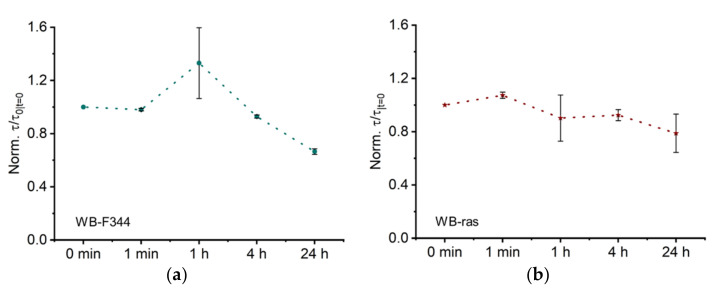
Trends in Cole parameters, characteristic time constant *τ*, over time following CAP treatment for two cell monolayers, WB-F344 (**a**) and WB-ras (**b**).

The characteristic time constant *τ* of the normalized Cole model for WB-F344 and WB-ras is shown in Figure 6a,b, respectively. At one hour, the *τ* values for WB-F344 increased dramatically to 1.3, although its error bar was significantly larger than for prior time points. *τ* recovered to a value close to 1 at 4 h and dropped to 0.7 after 24 h. The temporal development of *τ* for WB-ras cell monolayers demonstrated an overall steady drop with a slight rise at 1 min to 1.1. It subsequently declined progressively to 0.8 at 24 h.

The characteristic time constant *τ* of the Cole-Cole model represents the average time constants of the entire system. Its biological significance is not well understood but is widely believed to be related to the cell membrane capacitance [32]. However, our prior research studying the nsPEF-treatment of rat hepatocytes revealed that τ is influenced not only by cell membrane capacitance but also by the capacitance of intercellular connection regions [17,31]. Figure 6 shows that the change in *τ* values was greater in normal cells than in cancer cells, indicating that normal cells were more affected by CAP, which is consistent with the findings for *R_0_* versus *α*. In our previous study, it was observed that the change in *τ* of WB-F344 cell monolayers was larger than that of WB-ras cell monolayers after exposure to 100-ns pulsed electric fields [31]. Nevertheless, the differences in stimulation and interaction mechanisms necessitate more research into the potential effects of CAP on cell monolayers.

From a physical point of view, the Cole parameter *τ* reflects the average characteristic relaxation time of the system. In a recent study, where the distribution of dielectric relaxation times in cell monolayers were calculated, we observed that the change of *τ* was influenced by the variation of primary relaxation processes in the system [18]. In the frequency range between 100 Hz and 10 MHz, the primary polarization and relaxation processes in the cell monolayer are cell membrane interface polarization, which is influenced by the structure and composition of the cell membrane, the dielectric properties of the intracellular and extracellular environment, and the intercellular junctions [33]. The aforementioned discussion indicate that CAP treatment did not result in significant changes in the first two factors; therefore, it is plausible to hypothesize that alterations in intercellular junctions are the primary cause of the observed variations in *τ*. However, additional cross-validation experiments are required for a more thorough investigation of *τ*. Specifically, the parameter sensitivity in Section 2.1 revealed that *τ* is a low-sensitive parameter in the Cole-Cole model. The present optimization method may have significant errors when solving for *τ*. This low sensitivity makes biophysical interpretation of *τ* more challenging.

In Figure 4, Figure 5 and Figure 6, the large error bars in the Cole parameters after 1 h of CAP treatment may be comparable to our prior results for nsPEF-treatment [18] due to considerable changes in cell morphology. The principal dielectric relaxation processes in the cell monolayer, including the dielectric relaxation in different structures such as the cell-substrate, the cell itself, and intercellular junctions, changed with time at 1 h. This change over time may lead to substantial deviations in EIS measurements between different trials, which affects the fitting reproducibility for the Cole-Cole model.

#### 2.2.4. Potential Mechanisms of CAP Generated RONS Affecting Intercellular Junctions

The plasma source used in this study was the kINPen 11, which is a mature product with a well-calibrated performance [4]. Numerous investigations have demonstrated that the kINPen is capable of producing both long- and short-lived RONS in the media of similar experimental design [4,34]. Given the experimental conditions, such as nozzle to medium distance (10 mm), liquid depth (3 mm), and monolayer cells at the bottom, the contribution of short-lived species could be negligible. The kINPen produced a rather high concentration of H_2_O_2_ among the typical long-lived species in the culture medium, which could have significant effects on treated cells [35,36,37]. In addition, plasma-generated hydroxyl (OH) radicals can induce lipid peroxidation and disrupt cell membranes, which cannot be ignored. Furthermore, it has been demonstrated that the kINPen generated long-lived reactive nitrogen species (RNS) at lower concentrations compared to hydrogen peroxide (H_2_O_2_) and had insignificant effects on cells [38,39]. Therefore, the possible effects of H_2_O_2_ and OH are worth further exploration.

Numerous studies have examined the effect of H_2_O_2_ on intercellular junctions. According to a study by Haidari et al., H_2_O_2_ can preferentially disrupt E-calcium mucin and damage adherens junctions [40]. Inumaru and colleagues treated ARPE-19 cells with H_2_O_2_ and, discovered diminished expression of N-cadherin and dissociation of intercellular adhesion [41]. In addition, H_2_O_2_ can inhibit the production of Cx43, the primary gap junction protein, so disrupts the Gap junction [42]. Moreover, OH radicals can chemically react with the N terminus of the gap junction, destroying its structure [14]. On the other hand, CAP-generated ROS, such as H_2_O_2_ and OH radicals, can also damage the phospholipid bilayer structure of cell membrane, resulting in lipid peroxidation [43,44], decrease in membrane potential [45], and fragmentation of the membrane structure [46]. In summary, CAP treatment can undoubtedly change intercellular junctions. The lingering effects of long-lived ROS on membranes might explain why *τ* for WB-F344 cell monolayers remained unrecovered 24 h after CAP-treatment. However, the experimental results of this study were not sufficient to prove the hypothesis and further studies are needed.

## 3. Materials and Methods

### 3.1. Cell Culture

The WB-F344 cell line was derived from a normal adult male Fischer 344 rat liver by J.W. Grisham and colleagues [47]. The cancerous cell line WB-ras was derived by transfecting WB-F344 cells with the HRAS oncogene. As a result, these cells are characterized by the absence of contact inhibition, a spindle shape, and tumorigenicity *in vivo*. Both cell lines were obtained from Prof J E Trosko, Michigan State University, East Lansing, MI, USA. All Cells were cultivated in DMEM with 1 g/L glucose supplemented with 2 mM L-glutamine, 5% fetal calf serum (FCS) and 1% penicillin/streptomycin (all purchased from, Germany). Before impedance measurement the cells were cultured in ECIS-8W20idf-plates (Applied Biophysics, Inc., New York, NYC, USA) with 300 uL medium, ECIS-8W20idf with an integrated interdigitated electrode array were pre-treated with 10 mM L-cysteine 15 min before seeding cells to obtain a stable impedance. The experiments were conducted when confluent monolayers were confirmed 24 h after seeding by means of optical microscope.

### 3.2. CAP Treatments

**Figure 7:**
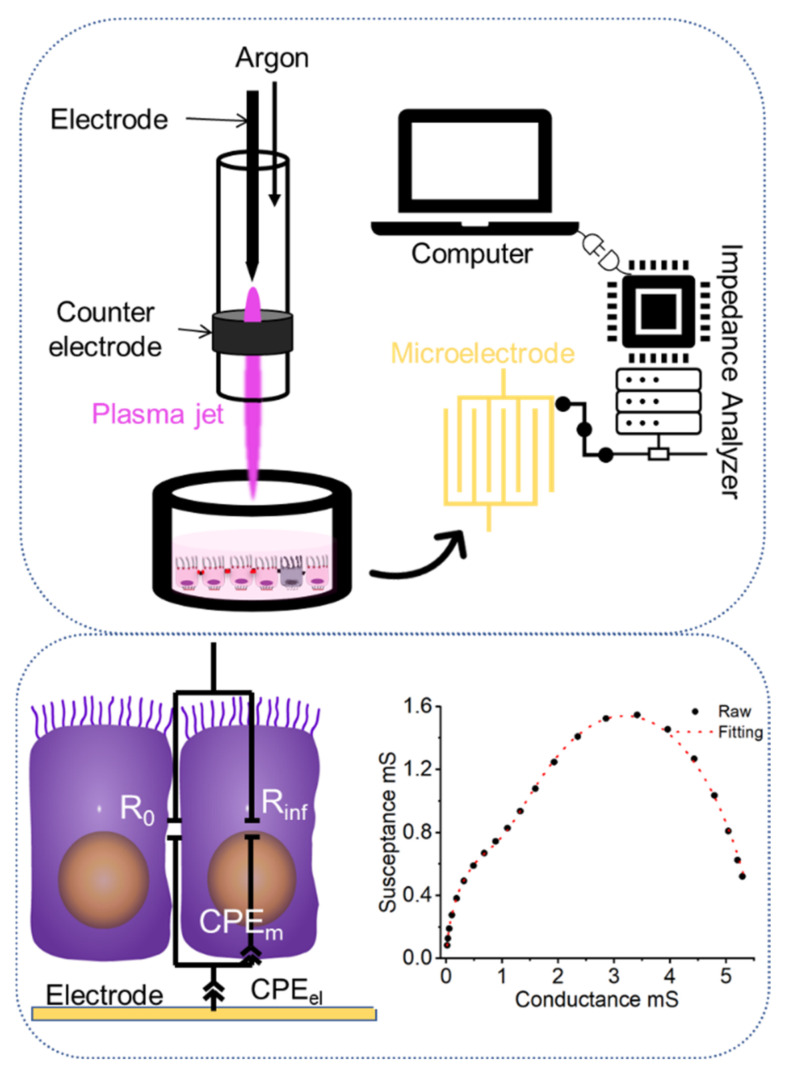
Schematic diagram for CAP treatments and impedimetric analysis.

The schematic diagram for the experimental setup is illustrated in Figure 7. kINPen 11 (Neoplas Control GmbH, Greifswald, Germany), a radiofrequency-driven (1.1 MHz, 6 kVpp) atmospheric pressure plasma jet, was utilized as the CAP source. Confluent cell monolayers were cultivated on top of microelectrodes in culture medium filled wells. The voltage and current waveforms, OES spectrum, temperature and more detailed characterization of the CAP source can be found in the literature [4,48]. High-purity (99.999%) argon gas was used as the working gas. The flow rate was set at 6 slm. The plasma nozzle was positioned 10 mm above the liquid level of the cell well to maximize treatment efficacy and minimize airflow’s influence [48]. The entire procedure is carried out at room temperature for 10 s.

### 3.3. Impedimetric Analysis

A detailed description of setup and procedures for the conducted bioimpedance measurements has been presented previously [17,31]. Basic steps and approaches are summarized in Figure 1.

In brief, impedance measurements were conducted from 100 Hz to 10 MHz before and after CAP-treatments. The ECIS chip was connected to an impedance analyzer (Agilent 4294A, Keysight Technologies, Inc., Santa Rosa, CA, USA) through the appropriate test fixture (Agilent 16047E, Keysight Technologies, Inc., Santa Rosa, CA, USA). Impedance of cell monolayers in each chamber was recorded in the computer and left for further analysis. Afterwards, EIS was analyzed by two Cole-models in series for the respective electrical components, i.e., describing electrode processes or monolayers:

(1) $$Z=Z_{ep}+Z_{cells}=\left[R_{infep}+\frac{R_{0ep}-R_{infep}}{1+{\left(i\omega \tau_{ep}\right)}^{\alpha_{ep}}}\right]+\left[R_{inf}+\frac{R_{0}-R_{inf}}{1+{\left(i\omega \tau \right)}^{\alpha }}\right]$$

The information of impedance spectra was summarized by four Cole parameters (*R_inf_*, *R_0_*, *α*, and *τ*). The first term in brackets represents electrode polarization; the term in the second bracket describes the contribution of the cell monolayer. *R_inf_* and *R_0_* represent the resistance at infinite frequency and at very low frequency, respectively. *(iωτ)^α^* is known as a constant phase element (CPE) to describe non-ideal capacitance, *Z_cpe_*, with *α* a dimensionless dispersion factor (0 < *α* < 1) and *τ* the characteristic time constant.

### 3.4. Uncertainty Quantification and Sensitivity Analysis

Uncertainty quantification can examine the effect of model input uncertainty on model output and improve model prediction accuracy. This study employs the chaotic polynomial method for UQ. The chaotic polynomial method regards the model as a black box and has the benefits of broad applicability and rapid computing. The preceding UQ analysis is calculable by numerical methods. Due to its simplicity and extensive applicability, the polynomial expansion method (PC) is frequently employed for this task. In this method, the original model *U* is expanded into a sequence of orthogonal polynomials, and the original model is approximated by a function of the input *Q*, as seen in the following expression [29]:

(2) $$U\approx \hat{U}\left(x,t,Q\right)=\overset{N_{p}-1}{\underset{n=0}{\sum }}C_{n}\left(x,t\right)\phi_{n}\left(Q\right)$$

where $\phi_{n}\left(Q\right)$ is the expanded polynomial, $C_{n}$ is the expanded coefficients, and $N_{p}$ is the number of expanded factors. The PC method approximates the original model with the proxy model U and leverages the orthogonality property and the zero-mean property to calculate the output uncertainty. The anticipated output value is roughly equivalent to the first coefficient of the polynomial expansion:

(3) $$E\left[Y\right]\approx E_{PC}\left[Y\right]=c_{0}$$

Similarly, the variance can be approximated by multiplying the sum of squares of known factors by the expansion coefficients:

(4) $$V\left[Y\right]\approx V_{PC}\left[Y\right]=\overset{N_{p}-1}{\underset{n=0}{\sum }}\gamma_{n}c_{n}^{2}$$

where $\gamma_{n}$ represents a normalization factor, denoted as $\gamma_{n}=E\left[\phi_{n}^{2}\left(Q\right)\right]$.

After calculating the mean and variance of the Cole-Cole model, the parameter sensitivity may be determined using the variance. SA is utilized to examine the magnitude of contribution for each model parameter to the model output uncertainty, hence simplifying the model and analyzing the relationship between the parameters. In this study, the Sobol index is utilized to investigate the influence of parameter changes in the Cole-Cole model on the real and imaginary parts of the output impedance. Sobol defined the Sobol index as a series of indices that permit the study of the interaction between all input parameters and their effect on the output [49]. The first order Sobol index is defined as the direct effect of each parameter on the model output variance:

(5) $$S_{i}=\frac{V\left[E\left[Y|Q_{i}\right]\right]}{V\left[Y\right]}$$

where $E[Y|Q_{i}]$ represents the expected value of the output *Y* while the input parameter is constant. Under the assumption that the interaction between parameters is not considered, the first-order Sobol index $S_{i}$ can be understood as the variance of the output *Y* induced by the input $Q_{i}$.

Calculating the higher-order Sobol indices one by one is a time-consuming process that allows for the calculation of interactions between distinct factors. Homma and Saltelli established the notion of total sensitivity index in order to consider the interactions between parameters without calculating all the higher order sensitivity indices [50]. This index takes into account the interaction between each parameter and the other parameters. Following is the calculation:

(6) $$S_{Ti}=1-\frac{V\left[E\left[Y|Q_{-i}\right]\right]}{V\left[Y\right]}$$

where $Q_{-i}$ denotes all uncertain parameters except $Q_{i}$. The total Sobol index, $S_{Ti}$, represents the overall variance caused by the input $Q_{i}$ and its interaction with the other parameters. If the first order Sobol index of a parameter is equal to the total Sobol index, interaction between this parameter and its other parameters can be disregarded.

As stated in Table 1, the Cole-Cole model parameters used for UQ and SA were adopted. The data were judged to be Gaussian-distributed from the results of impedance spectrum fitting for WB-F344 cell monolayers in this and previous experiments [17,19,31], and the mean values and variation ranges were determined.

## 4. Conclusions

This study investigated CAP treatment on a rat liver epithelial cell (WB-F344) and its malignant counterpart (WB-ras) using impedance spectroscopy followed by analysis with the Cole-Cole model. According to experimental impedance measurements of the cell monolayer, the Cole-Cole model was investigated using UQ and SA. Under specified situations, the results revealed that UQ can determine the frequency cut-off point between low and high frequency resistances. We found that *R_0_* and *α* were the most sensitive parameters, while *R_inf_* and *τ* were the least sensitive. The temporal development of Cole parameters suggests that CAP induced reversible changes in intercellular junctions but did not cause significant changes in membrane permeability. Long-term alterations in the cell monolayer after CAP-treatment suggest that long-lived ROS, such as H_2_O_2_, might play a nonnegligible role. The results of this study may provide some insight into a better understanding of how CAP interacts with intercellular junctions. The UQ and SA proposed for the analysis of Cole parameters could also guide the evaluation of other methods of exposure. Given the broad utility of the Cole model, continuation of this research on biological tissues will be a topic of our interest.

## Figures and Tables

**Table 1:** Parameters of Cole-Cole model for UQ and SA.

Parameters	Mean Values	Sigma ^1^
*R_inf_*	200 Ω	60 Ω
*R_0_*	1000 Ω	300 Ω
*α*	0.7	0.21
*τ*	5 × 10^−6^ s	1.5 × 10^−6^ s

^1^ Sigma represents the standard deviation for the Gaussian function.

## Data Availability

The data presented in this study are available on request from the corresponding author.

## Sample Availability

Samples of the compounds are available from the authors.
